# Assessing Developmental Environmental Risk Factor Exposure in Clinical High Risk for Psychosis Individuals: Preliminary Results Using the Individual and Structural Exposure to Stress in Psychosis-Risk States Scale

**DOI:** 10.3390/jcm8070994

**Published:** 2019-07-09

**Authors:** Teresa Vargas, Denise S. Zou, Rachel E. Conley, Vijay A. Mittal

**Affiliations:** 1 Department of Psychology, Northwestern University, Evanston, IL 60208, USA; 2 Department of Psychology, Department of Psychiatry, Department of Medical Social Sciences, Institute for Innovations in Developmental Sciences, Institute for Policy Research, Northwestern University, Evanston, IL 60208, USA

**Keywords:** development, chronic stress, environmental stress, cumulative environmental risk factors, environmental risk factors, clinical high risk, psychosis, developmental stress

## Abstract

Introduction: Exposure to cumulative environmental risk factors across development has been linked to a host of adverse health/functional outcomes. This perspective incorporating information regarding exposure at differing developmental periods is lacking in research surrounding individuals at Clinical High Risk (CHR) for developing a psychotic disorder. Methods: CHR individuals (*n* = 35) and healthy volunteers (*n* = 28) completed structured clinical interviews as well as our group’s newly developed Individual and Structural Exposure to Stress in Psychosis-risk-states (ISESP) interview. Lifetime cumulative scores were calculated, and severity of stress was reported for multiple developmental periods/ages. Group differences were tested, and associations with current symptom domains were examined. Results: Significant group differences were not observed for lifetime cumulative events, though CHR trended toward endorsing more events and greater stress severity. For stress severity across development, there were trending group differences for the 11–13 age range, and significant group differences for the 14–18 age range; notably, comparisons for earlier time points did not approach statistical significance. Associations between negative symptoms and cumulative severity of exposure were observed. Discussion: Results suggest exploring exposure to cumulative environmental risk factors/stressors and stress severity across developmental periods is generally informative and possibly specifically so for predictive models and diathesis-stress psychosis risk conceptualizations.

## 1. Introduction

Exposure to environmental factors may increase risk for developing psychopathology in those with a pre-existing vulnerability, through exacerbating chronic stress exposure [1,2]. This is especially impactful when exposure occurs during critical neurodevelopmental periods [3,4]. Though this is well understood, scales assessing exposure to environmental stressors during development are scarce for Clinical High Risk for Psychosis (CHR) populations. Gaining a more thorough understanding of exposure to chronic stressors during key developmental periods is a critical future direction for enriching measurement of risk for developing psychopathology in this population [5,6,7,8,9]. Ultimately, it is a necessary future direction for targeting prevention and intervention efforts.

The presence of environmental risk factors (e.g., bullying, financial or family instability, exposure to violence, death of a loved one, etc.) has been consistently associated with a host of adverse health and functional outcomes [1,2,10,11,12,13,14,15,16]. Within the psychosis spectrum literature, undergoing chronic environmental risk factors is also reported to relate to symptoms, illness severity, and functional outcomes [5,7,17,18,19,20,21,22,23,24]. A central proposed mechanism for this relation is chronic exposure to stress compromising neural systems underlying affect regulation and executive function, along with neuroendocrine systems including the Hypothalamic Pituitary Adrenal (HPA) axis [23,25,26,27,28,29]. Given the marked putative association between environmental risk exposure and symptom progression in psychosis, it is critical to further understand its role prior to psychotic illness onset during the prodromal stage. This period happens during adolescence and young adulthood and is marked by attenuated psychotic symptoms and accompanying functional impairment. Individuals at CHR for psychosis (those meeting for a psychosis risk syndrome) include a large subgroup of those in the prodromal stage—as many of 10–30% transition to a psychotic disorder within a two-year period [9,30,31]. More fully clarifying the influence of environmental risk exposure at this stage of illness progression could allow us to understand relations to etiology free of confounds intrinsic to chronic psychotic illness, such as medication use. This neural diathesis approach conceptually dating back to Meehl’s theory of schizotypy is bound to aid in elucidating adverse life experiences that, together with subclinical/attenuated symptomatology, may precipitate a clinical psychotic state [32]. Further, adopting this approach could be useful in informing prevention and intervention models to ultimately prevent transition to a psychotic disorder in at-risk individuals [8].

In terms of measuring exposure to environmental risk factors, allostatic load and aggregate dysregulation models of stress exposure suggest there are strong benefits in studying cumulative risk (multiple environmental risk factors/stressors at once, rather than individually) [1,13,33]. The concept behind this model is that types of risk exposure may have similar developmental consequences [1,13,33]. In addition, differing environmental risk exposure may compromise converging biological systems, including affect regulation and executive function neural systems [34]. Yet, psychosis risk models of psychopathology often focus on single risk factors or stressors, such as childhood trauma [30,31]. This is despite the notion that exposure to more than one environmental risk factor yields more adverse developmental consequences. For example, according to the National Center for Children in Poverty, in 2010 20% of American children under 6 years of age had experienced 3 or more developmental risk factors, and 41% had experienced 1–2 risks [13]. Compared to single stressor/risk factor models, cumulative environmental risk models are also beneficial in that they avoid problems surrounding overestimation of the impact of a singular risk factor (which may often be correlated with exposure to other risk factors in an individual) [13]. Lastly, these models have also been found to be more powerful, in addition to offering an increased prospective prediction advantage [3,13,34]. Thus, understanding environmental effects on symptoms in CHR individuals may be essential for informing degrees of risk of future illness.

Another critical factor to consider is that of development during the adolescent and young adult stage coinciding with the CHR period. This developmental stage is characterized by dynamic brain maturation and plasticity, which makes it more liable to be sensitive to exposure to environmental risk factors and stressors [3,4,35]. For example, the prefrontal cortex, which underlies essential stress and affect regulation functions, does not fully mature until the third decade of life, and is therefore undergoing significant development during this stage [3,34]. Emerging studies in non-clinical populations indicate that examining stressors across differing stages of development is crucial [4,6,9,13,14,27,36]. However, the CHR literature has seldom examined stressors cumulatively, and it has seldom examined aggregate stressors across differing stages of development. More fully considering this facet of environmental risk exposure could award us etiological clues, as well as ultimately supplement risk models for conversion to a psychotic disorder.

We developed a scale to address these gaps in the literature. The Individual and Structural Exposure to Stress in Psychosis risk states (ISESP) scale was compiled to capture cumulative exposure to environmental risk factors/stressors, as well as for isolating during which stages of development these stressors were occurring in an individual. A unique facet of the scale is that it captures exposure to both individual-level stressors (such as family stressors, exposure to bullying) and structural-level stressors (such as neighborhood characteristics and socioeconomic status). The scale is comprised of a comprehensive checklist of lifetime events constituting environmental risk factors, which the participant fills out with the help of a clinician, among other written questions. Afterward, the participant undergoes a thorough interview with a trained clinician assessing the nature of the exposure to each event, along with time period and severity of exposure. Crucially, the scale was developed in order to maximize retrospective recall of stressors, though implementing techniques to enhance salience and contextual detail surrounding the events (one example of this is probing for proxy socioeconomic status measures that are more readily remembered, i.e., “number of rooms in the home” vs. “annual income”) [37,38]. With regards to structural environmental risk factors, the scale also contains measures that can objectively assess exposure to risk (i.e., acquiring zip codes that can then be used to extract crime data for that region in order to determine participant environmental exposure to violence).

The current study presents preliminary data for the ISESP. First, cumulative endorsement of environmental stressors was compared across CHR and healthy volunteers. The first aim was to test whether stressful life events were more prevalent in CHR. The first hypothesis sought to replicate meta-analytic evidence finding traumatic events and recent stressful events to be more prevalent in CHR individuals [39,40]. Exposure to environmental risk factors was measured in terms of exposure and self-reported severity of exposure. It was thus hypothesized that CHR individuals would endorse more lifetime events, and greater severity of lifetime events. The second aim was to build on the existing literature by incorporating the nuance of exposure during distinct developmental periods, incorporating self-reported severity of exposure. Given the present gap in the literature regarding this question, we remained agnostic with regards to hypotheses, though we expected greater endorsement of chronic stress in CHR overall. Finally, the third aim was to relate environmental risk/stressor variables to functional outcomes, namely positive and negative symptoms, as well as tolerance to normal stress and dysphoric mood. It was hypothesized that environmental risk variables would be associated with symptoms, such that greater environmental risk predicted greater symptoms. Additionally, an exploratory aim was to relate positive and negative symptomatology to stress severity during the differing developmental time points (determined by participant’s age at time of exposure).

## 2. Participants

Participants included 62 adolescents and young adults (34 CHR and 28 healthy volunteers) recruited to the Adolescent Development and Preventive Treatment (ADAPT) research program at Northwestern University, including Chicago and surrounding suburbs. All procedures were approved by the University Institutional Review Board (STU00203263). The CHR sample was entirely antipsychotic-naïve. Ages 16–30 were eligible for inclusion. CHR individuals were included if they met criteria for clinical high risk according to the Structured Interview for Psychosis risk Syndromes (SIPS). Healthy volunteers were included provided they did not meet criteria for a CHR syndrome and did not have first-degree relatives diagnosed with a psychotic disorder. Exclusion criteria for both CHR and healthy volunteers included history of head injury, history of a neurological disorder, and having an Axis I psychotic disorder. Participants 18 and older provided written informed consent. For participants under 18, parents or legal guardians additionally provided consent.

## 3. Methods

**Figure 1:**
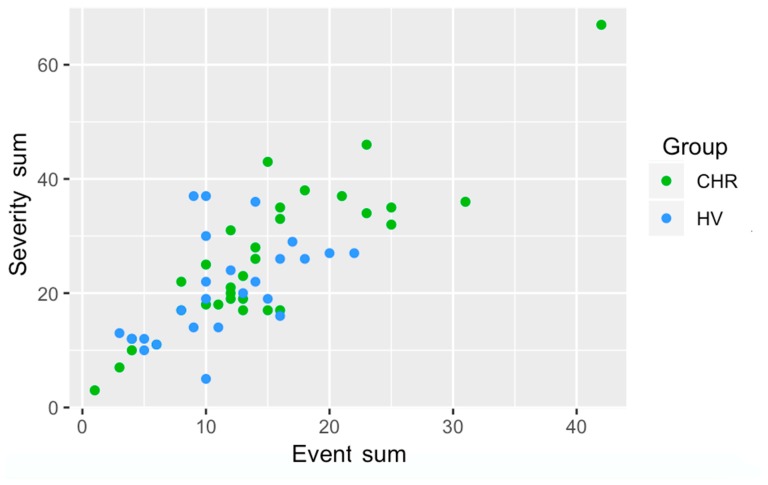
Number of events endorsed and sum of severity of endorsed events by group, CHR = Clinical High Risk, HV = Healthy Volunteer.

**Figure 2:**
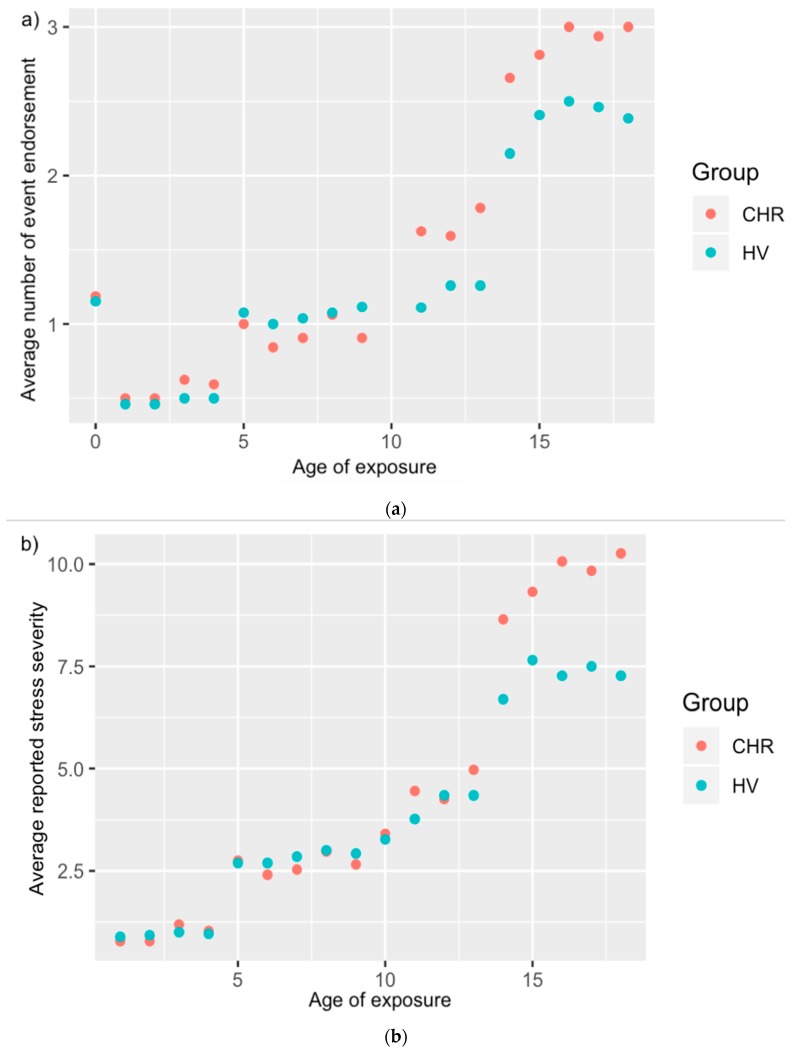
Average number of event endorsement (**a**) and stress severity rating (**b**) per age of exposure per group.

*Environmental Risk Interview.* The Individual and Structural Exposure to Stress in Psychosis risk states (ISESP) interview was administered to assess exposure to individual and structural environmental risk. This scale was partially adapted from the Family Socialization Interview (FSI), which assesses exposure to stressors including family changes, partner/guardian changes, parent–child separation, job/finances, housing, illness/injury, deaths of loved ones, and legal, among other stressors [41]. In addition to these stressors, items were added related to, for example, feelings of environmental lack of safety (at school and at home), exposure to interpersonal stressors such as bullying, lack of feeling of belonging among peers, feelings of rejection, lack of close relationships, academic problems, and more general problems at school. In addition to the focus of the current preliminary investigation, the scale also has components related to objective measures of structural risk exposure (i.e., home neighborhood crime exposure and school neighborhood crime exposure) as well as measures of socioeconomic status, among other individual environmental risk factors. Participants were interviewed by a doctoral student about each individual stressor, in order to collect information on timing of exposure, as well as severity of exposure. Severity of exposure for each stressor was coded on a Likert scale of 1–5, with 1 signifying “happened but not stressful,” and 5 signifying “very stressful.” Events and reported severity were summed per participant (see Figure 1). Exposure information was coded per age of the participant (see Figure 2). Finally, participants were asked to make cumulative ratings of how stressful differing time periods had been overall. In order to facilitate recall of retrospective information, these periods were broken up to roughly coincide with periods of schooling for anchoring purposes [37]. There were four periods, and these were anchored based on schooling stage, with 0–4 linked to preschool, 5–10 linked to elementary school, 11–13 linked to middle school, and 14–18 linked to high school. Participants were asked to recall that period (e.g., 5–10), and then told that this period would roughly coincide with elementary school years in order to aid recall. Participants were then asked to specify how stressful they had found that age period overall, on the same Likert 1–5 scale used for individual stressors. This question was asked at the very end, after participants had discussed each event exposure in detail, in order to facilitate better retrospective recall. For these analyses of developmental time points, due to missing data, the resulting sample size was 24 CHR and 20 HV.

*Structured Clinical Interviews and Assessment.* The Structured Clinical Interview for *DSM-IV* Axis I disorders (SCID) was administered to assess history of mood and anxiety disorders, as well as to rule out psychosis diagnoses in the CHR and HV groups [42]. The SIPS was administered to rule out symptoms in HVs, identify CHR subjects and measure symptoms [43]. Interviews were conducted by trained clinical doctoral students; Kappas of at least 0.8 for SIPS and 0.9 for psychosis risk and psychiatric diagnoses were obtained.

*Analytic Strategy.* Analyses were run using SPSS Version 25. Independent samples Mann–Whitney U-tests and chi-square tests of independence, where appropriate, were used to examine demographic differences characteristics (age, sex, race) between CHR and HV groups. Z tests were used (Skewness/SE_skewness_ and Kurtosis/SE_kurtosis_) to evaluate normality of continuous variables, with z values of > |1.99| considered to be evidence of non-normal distribution [44]. Non-parametric testing was conducted for all analyses, due to evidence of skew in the symptom and environmental risk data. Total number of events endorsed over the lifetime and sum of severity of stress per endorsed event were summed, and Independent Samples Mann–Whitney U tests were conducted to examine group differences. Group differences in self-reported severity of stress exposure during the 0–4, 5–10, 11–13, and 14–18 age ranges were also examined. Finally, Spearman correlations between symptoms, total sum of events endorsed and total sum of severity of events endorsed were conducted in the CHR group. In addition, exploratory Spearman correlations were conducted between self-reported stress severity during the 4 developmental time periods (0–5, 5–10, 11–13, 14–18), positive and negative symptoms.

## 4. Results

*Demographics, skewness and kurtosis for target variables.* There were no differences between groups in age or race. There were more females in the healthy volunteer group (see Table 1). The 0–4, 5–10, and 14–18 periods, event sum, and event severity all exhibited significant levels of skewness as determined by Z values greater than |1.99|. Only the 0–4 age range, event sum, and event severity exhibited significant levels of kurtosis as determined by Z values greater than |1.99| (see Table 2).

*Group differences in event exposure and severity.* The independent samples Mann–Whitney U Test revealed a trending effect of CHR individuals endorsing more events, *p* = 0.12, with sum of severity of events endorsed yielding a non-significant association, *p* = 0.18 (see Figure 1).

**Figure 3:**
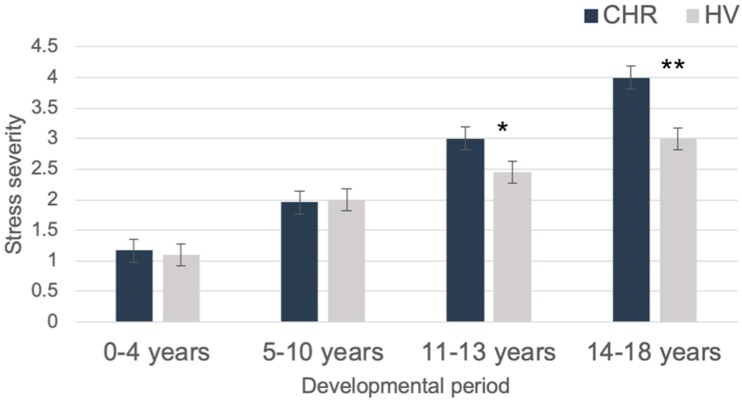
Stress severity per developmental time period by group. *Note*. * = *p* ≤ 0.05, ** *p* < 0.01.

*Group differences in stress exposure at differing developmental timepoints.* The independent samples Mann–Whitney U Test did not reveal significant group differences for severity of stress exposure during the 0–4 age period (*p* = 0.89), or the 5–10 age period (*p* = 0.93). There was a group difference nearing significance for the 11–13 age period (*p* = 0.05, *η*^2^ = 0.09), such that CHR individuals reported more severe stress exposure during this time range (see Table 3). Finally, there was a significant group difference for the 14–18 time range (*p* = 0.003, *η*^2^ = 0.21), such that CHR individuals reported more severe stress exposure during this time range, compared to healthy volunteers (see Figure 3).

**Figure 4:**
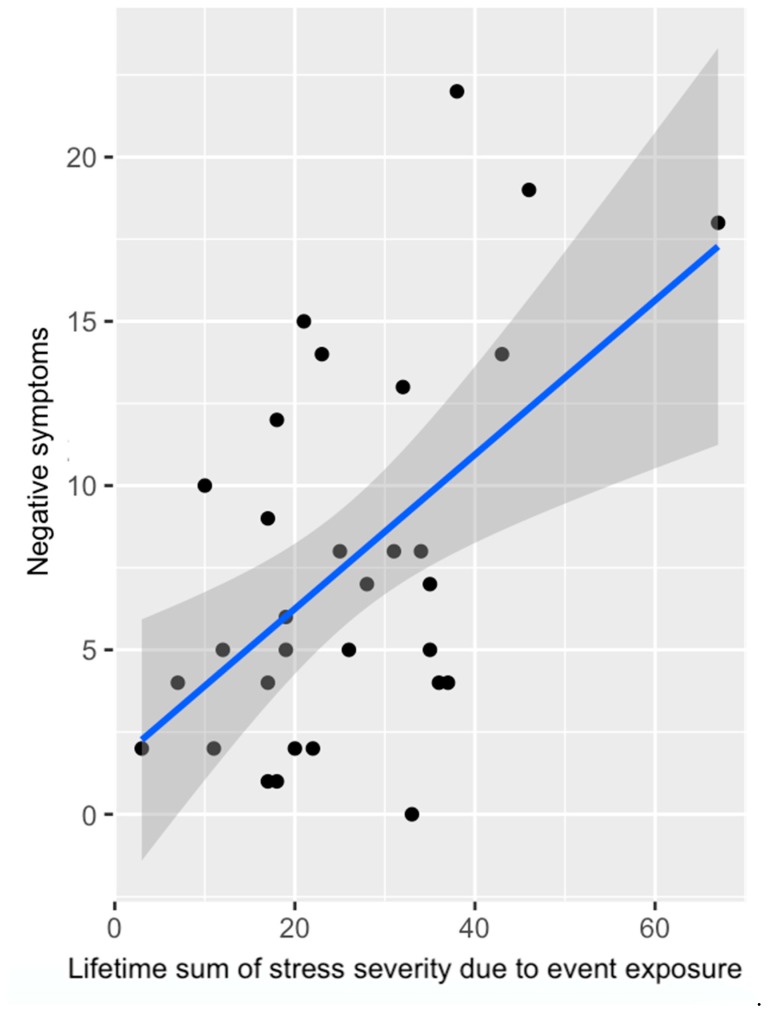
Association between negative symptoms and reported severity of stress due to event exposure in CHR individuals; for analyses, variables were rank-ordered due to skew in the data (Spearman *r* = 0.36, *p* = 0.048).

*Associations with symptoms.* Within the CHR group, there was no association between positive symptoms and total number of events endorsed (Spearman *r* = 0.11, *p* = 0.52). However, greater number of events endorsed trended toward being associated with greater negative symptoms (*r* = 0.29, *p* = 0.09). With regards to reported severity of stress due to event exposure, the relation between positive symptoms and severity of stress exposure did not meet criteria for statistical significance (Spearman *r* = 0.24, *p* = 0.19). Finally, there was a significant association between reported severity of stress due to event exposure and negative symptoms (Spearman *r* = 0.36, *p* = 0.048) (see Figure 4). For total stress severity, greater dysphoric mood was associated with greater total reported severity (*r* = 0.38, *p* = 0.036). Likewise, more impaired tolerance to normal stress was associated with greater reported stress severity (*r* = 0.54, *p* = 0.002).

Exploratory associations between severity of stress exposure at differing developmental time points and symptoms within CHR individuals. Within the 0–4 time range, there were no significant associations with positive (Spearman *r* = 0.21, *p* = 0.35) or negative (Spearman *r* = 0.03, *p* = 0.89) symptoms. Within the 5–10 age range, there were no significant associations with positive (Spearman *r* = 0.14, *p* = 0.53) or negative (Spearman *r* = 0.1, *p* = 0.67) symptoms. Within the 11–13 age range, there were no significant associations with positive (Spearman *r* = 0.12, *p* = 0.57) or negative (Spearman *r* = 0.01, *p* = 0.97) symptoms. Within the 14–18 age range, there was a trending association between positive symptoms and severity of stress exposure (Spearman *r* = 0.41, *p* = 0.05). In this age range, there was no significant association between negative symptoms and severity of stress exposure (Spearman *r* = 0.16, *p* = 0.48).

## 5. Discussion

The current investigation presents preliminary data for the ISESP scale. CHR individuals trended toward endorsing a greater total number of events. These results are partially consistent with the literature, which has not always found overall differences in endorsement of stressful life events in CHR [39,40,45]. Interestingly, there were group differences in severity of chronic stress exposure for the 14–18 age range, and nearing significance (*p* = 0.05) for the 11–13 age range, such that CHR individuals reported greater chronic stress severity during these developmental time points. Group differences were not observed for the 0–4 and 5–10 age ranges. Taken in light with a with neural diathesis conceptualization of psychosis risk [9,31,46], these results may suggest that exposure to environmental risk and chronic stress during differing developmental periods may be an important future direction for the field. Finally, there were significant associations such that greater lifetime stress severity was associated with greater negative symptoms, and trending associations were also observed with total number of endorsed events. Exploratory analyses of stress severity and symptomatology in differing developmental periods also found a strong trend between positive symptoms and reported stress severity during the latest developmental period. These results suggest that measurement of both environmental risk exposure and reported stress severity is pertinent to CHR symptomatology. While more power is necessary to appropriately test the value of a cumulative score, the current preliminary study found strong results to support the value of focusing on different developmental time points [3,6,13,28].

Results concerning total event endorsement and stress severity were in the direction of, but did not meet significance criteria to support original hypotheses, as meta-analytic evidence has shown that CHR individuals endorse a greater proportion of traumatic events and recent stressful events [39]. In the present sample, CHR individuals had greater average total event endorsement, as well as greater average severity reported, though the group difference did not meet threshold for statistical significance. This is not altogether surprising, given that the literature has not been conclusive with regards to endorsement of environmental factors conferring sources of chronic stress [40]. For example, the literature constituting stressful life events (defined as dangerous or life-changing experiences occurring for an individual) has thus far proven inconclusive with regards to CHR endorsement compared to healthy volunteers [40]. Increased number of current endorsed life events has been found in CHR individuals [45]; however, this is distinct from our cumulative measure of total exposure across the lifetime. In all, results suggest that accumulating stressors across the lifetime could be a less sensitive method of understanding an individual’s exposure to environmental risk factors and chronic stress.

The ISESP scale was designed to create more specificity in this regard, by collecting information on timing of exposure and severity across the lifetime. After a detailed interview of each event endorsed, participants were asked to rate stress severity during differing developmental time points. Results showed that though there were no differences in stress severity ratings for 0–4 and 5–10 age ranges, there was a group difference nearing significance (*p* = 0.05) such that CHR individuals endorsed greater stress severity during the 11–13 age range, and there was also a significant group difference such that CHR individuals endorsed greater stress severity during the 14–18 age range. Observed results are fully consistent with neural-diathesis conceptualizations of developing psychotic disorders [9,40,47]. An underlying diathesis, or pre-existing vulnerability (e.g., genetic, biological), may interact with the increasing demands of emerging adolescence and adulthood to increase risk for developing psychopathology. Indeed, during these critical stages that we observed group differences in, the early and later adolescent (i.e., 11–13 and 14–18) ranges, there is a confluence of endogenous developmental processes that, if altered, may confer increased vulnerability and present greater sensitivity to environmental influence [47]. The prefrontal cortex, which continues normative maturation until the third decade of life, possesses a high density of stress-susceptible glucocorticoid and dopaminergic projections [3]. During these time periods, there are also functional alterations of the HPA axis and sympathetic nervous system (SNS), which could lead to neuroimmune and corticotrophin abnormalities in vulnerable populations exposed to environmental risk factors during these time periods [3,29,36,47,48,49,50].

Thus, examining stress severity and environmental risk factor exposure during these developmental time points could provide us with valuable information that would be missed when undertaking a cumulative approach across the whole lifetime, or a cumulative approach of recent or current exposure to environmental risk factors and chronic stress. Perhaps exposure during a particular developmental time point is most impactful overall; or perhaps depending on what one is trying to predict (e.g., executive function versus social function versus symptomatology), exposure to chronic stress and environmental risk factors during certain developmental time points becomes more or less relevant. It will be crucial for future studies to further delve into this matter by further incorporating a developmental perspective with regards to both environmental risk exposure and chronic stress exposure. Of course, it is also possible that due to the retrospective nature of the ISESP, events that happened earlier in the participant’s life (0–10 age ranges) were harder to recall, and so there were some omitted events that influenced the results in both groups. Relatedly, an important future direction in scale development is to incorporate optional confirmation data points into the scale, so that the self-reported information can be cross-checked with hospital records, in addition to information from treatment providers and relatives/significant others.

Finally, the present investigation sought to determine whether environmental risk exposure and reported stress severity variables would be associated with symptomatology in CHR individuals. Trend level associations were observed with cumulative events endorsed and negative symptoms. Interestingly, there were also significant associations between reported stress severity and negative symptoms. Further, associations between reported stress severity and positive symptoms were observed in the 14–18 age range in exploratory analyses (*r* = 0.41). These results support the literature that strongly suggests environmental stressors are relevant to symptomatology and illness progression [19,39,40,46,51,52,53]. Further, stress severity was correlated with both dysphoric mood and impaired tolerance to normal stress. This result lends credence to the notion that measuring environmental risk is beneficial in assessing broader risk beyond the psychosis spectrum. Taken together, preliminary data suggests that taking a cumulative approach across development is a valuable and informative avenue that has thus far not received as much attention in the CHR literature, and so the present results create avenues for promising future directions of study in this area.

Though current results and conceptualizations are promising, there are certainly limitations to keep in mind and tamper interpretations. First and foremost, the limited sample size of the current study means that results, though promising, ought to be interpreted as preliminary. It will be paramount to re-visit these questions in larger samples in order to corroborate present findings. The ISESP was developed keeping in mind the limitations of retrospective reporting, and several strategies were adapted in order to minimize inaccuracies around retrospective recall, including using techniques such as anchoring, and increasing contextual detail and specificity whenever possible [37,38]. Nonetheless, it would be ideal to collect longitudinal data, or to corroborate reports with significant others and family. Thus, this is an important future direction.

The new ISESP scale was compiled from validated scales and methods. However, it will be critical to establish formal validity and reliability for the ISESP scale once data collection has progressed further and sample sizes are larger. Future, more well powered samples would also benefit from collecting and comparing data across stages of psychotic illness progression, including non-clinical psychosis and chronic psychosis cases. This would aid in obtaining a fuller picture of psychotic disorder etiology from a diathesis-stress perspective. It would also be highly pertinent to address questions regarding biological mechanisms, by using cumulative exposure and stress severity to predict neural function/structure across development. Though there remain components of the ISESP scale that have yet to be presented, the current study represents important first steps toward determining its utility in measuring risk exposure in CHR populations. Taken together, results are promising, though they ought to be taken as preliminary, and future studies with larger samples will be needed in order to corroborate present findings.

## 6. Conclusions

In conclusion, the present investigation supports the notion that developmental timing of exposure to chronic stressors could be critical for understanding psychopathology risk. In our sample, differences in severity of stress exposure between HV and CHR became apparent only during the 11–13, and 14–18 age range. These periods coincide with critical developmental timepoints, including frontal and corticolimbic neurodevelopment underlying executive and affective functions [3]. Alterations in these executive and effective functions could in turn be relevant for symptom presentation. Severity of stress exposure overall was related to negative symptoms, preliminarily suggesting that negative symptomatology may reflect this fact, and meaningfully relate to psychobiological correlates of stress exposure. In all, results suggest that it will be critical for future studies to explore specificity of affected cognitive and affective functions, as this relates to age of exposure and developmental timing of these stressors. Ultimately, this could inform models of environmental risk for developing psychopathology, and aid in prediction and intervention efforts.

## Figures and Tables

**Table 1:** Demographic Characteristics.

	HV *n* = 28 (1)	CHR *n* = 35 (2)	Group Diff.
**Demographics**			
Sex (Female/Male)	22/6	18/17	1 > 2 *
Age Mean (SD)	20.04 (2.12)	20.63 (1.91)	1 = 2
**Race**			1 = 2
First Nations Count (%)	1 (3.6%)		
East Asian Count (%)	4 (14.3%)	3 (8.6%)	
Southeast Asian Count (%)	3 (10.7%)		
South Asian Count (%)	0	2 (5.7%)	
Black Count (%)	4 (14.3%)	10 (28.6%)	
Central/South American Count (%)	1 (3.6%)	3 (8.6%)	
West/Central Asia, Middle East Count (%)	1 (3.6%)	0	
White Count (%)	11 (39.3%)	14 (40%)	
Interracial Count (%)	3 (10.7%)	3 (8.6%)	
Hispanic (no/yes)	25/3	26/9	1 = 2
**Symptoms**			
Positive ^a^ Mean (SD)	0.77 (1.45)	11.97 (3.50)	1 < 2 *
Negative ^a^ Mean (SD)	0.87 (1.58)	7.91 (6.07)	1 < 2 *

* *p* < 0.05. ^a^ Measured by Structured Interview for Psychosis-Risk Syndromes (SIPS) battery.

**Table 2:** Skewness and kurtosis for Individual and Structural Exposure to Stress in Psychosis-Risk States (ISESP) target variables.

Variable	Skewness	SE_skewness_	Kurtosis	SE_kurtosis_
Event sum	1.29	0.302	3.065	0.595
Event severity	1.023	0.314	2.259	0.618
Developmental time points				
0–4 age range	3.87	0.357	20.07	0.702
5–10 age range	0.803	0.357	0.821	0.702
11–13 age range	0.073	0.357	0.447	0.702
14–18 age range	−0.811	0.357	0.223	0.702

**Table 3:** Characteristics of event endorsement and stress severity.

	HV (1)	CHR (2)
**Event endorsement ^a^**		
Mean	11	14.37
Median	10	13
SD	4.98	8.57
Range	19	41
Minimum	3	1
Maximum	22	42
**Reported stress severity ^b^**		
Mean	20.65	25.53
Median	19.50	22.5
SD	8.82	13.04
Range	32	64
Minimum	5	3
Maximum	37	67
**Stress severity per time period ^c^**		
*Preschool (~0–4 years)*		
Mean	1.10	1.17
Median	1.00	1
SD	0.31	0.64
Range	1	3
Minimum	1	1
Maximum	2	4
*Elementary School (~5–10 years)*		
Mean	2	1.96
Median	2	2
SD	1.03	0.96
Range	4	3
Minimum	1	1
Maximum	5	4
*Middle School (~11–13 years)*		
Mean	2.45	3
Median	2	3
SD	0.76	0.93
Range	3	3
Minimum	1	2
Maximum	4	5
*High School (~14–18 years)*		
Mean	3	4
Median	3	4
SD	1.08	1.18
Range	4	4
Minimum	1	1
Maximum	5	5

^a^ Total number of events endorsed were added per subject, resulting in overall event endorsement count. ^b^ Severity per event endorsed was rated from 1 to 5, 5 being “Very stressful”, and 1 being “Happened but not stressful.” Severity ratings for each event endorsed were added per subject, resulting in overall reported stress severity. ^c^ Rated from 1 to 5, 5 being “Very stressful”, and 1 being “Happened but not stressful.”.
